# Current practice of colonoscopy surveillance in patients with lynch syndrome: A multicenter retrospective cohort study in Japan

**DOI:** 10.1002/deo2.179

**Published:** 2022-11-01

**Authors:** Yasuyuki Miyakura, Akiko Chino, Kohji Tanakaya, Alan Kawarai Lefor, Kiwamu Akagi, Akinari Takao, Masayoshi Yamada, Hideyuki Ishida, Koji Komori, Kazuhito Sasaki, Masashi Miguchi, Keiji Hirata, Tomoya Sudo, Toshiaki Ishikawa, Tatsuro Yamaguchi, Naohiro Tomita, Yoichi Ajioka

**Affiliations:** ^1^ Department of Surgery Saitama Medical Center Jichi Medical University Saitama Japan; ^2^ Department of Surgery Jichi Medical University Tochigi Japan; ^3^ The Committee of Hereditary Colorectal Cancer, Japanese Society for Cancer of the Colon and Rectum Tokyo Japan; ^4^ Department of Gastroenterology, Cancer Institute Hospital Japanese Foundation for Cancer Research Tokyo Japan; ^5^ Department of Surgery Iwakuni Clinical Center Yamaguchi Japan; ^6^ Department of Molecular Diagnosis and Cancer Prevention Saitama Cancer Center Saitama Japan; ^7^ Department of Gastroenterology Tokyo Metropolitan Cancer and Infectious Diseases Center Komagome Hospital Tokyo Japan; ^8^ Endoscopy Division National Cancer Center Hospital Tokyo Japan; ^9^ Department of Digestive Tract and General Surgery Saitama Medical Center, Saitama Medical University Saitama Japan; ^10^ Department of Gastroenterological Surgery Aichi Cancer Center Hospital Aichi Japan; ^11^ Department of Surgical Oncology, Faculty of Medicine The University of Tokyo Tokyo Japan; ^12^ Department of Surgery Hiroshima Prefectural Hospital Hiroshima Japan; ^13^ Department of Surgery I, School of Medicine University of Occupational and Environmental Health Fukuoka Japan; ^14^ Department of Surgery Kurume University Fukuoka Japan; ^15^ Department of Medical Oncology, Faculty of Medicine Juntendo University Tokyo Japan; ^16^ Department of Clinical Genetics, Tokyo Metropolitan Cancer and Infectious Diseases Center Komagome Hospital Tokyo Japan; ^17^ Cancer Treatment Center Toyonaka Municipal Hospital Osaka Japan; ^18^ Japanese Society for Cancer of Colon and Rectum Tokyo Japan; ^19^ Division of Molecular and Diagnostic Pathology, Graduate School of Medical and Dental Sciences Niigata University Niigata Japan

**Keywords:** adenoma detection rate, colonoscopy surveillance, complication, endoscopic interval, Lynch syndrome

## Abstract

**Objectives:**

Colonoscopy surveillance reduces the incidence of colorectal cancer through the detection and endoscopic removal of adenomas. Current guidelines recommend that patients with Lynch syndrome should have colonoscopy surveillance every 1–2 years starting at the age of 20–25. However, insufficient data are available to evaluate the quality and safety of colonoscopy surveillance for patients with Lynch syndrome nationwide in Japan.

**Methods:**

Patients with Lynch syndrome (*n* = 309) from 13 institutions who underwent one or more colonoscopy procedures were enrolled in this retrospective analysis. Colonoscopy completion rate, colonoscopy‐related complication rate, proportion with an adequate colonoscopy interval, and adenoma detection rate were reviewed.

**Results:**

The colonoscopy completion rate was 98.8% and a history of previous colorectal cancer surgery was significantly associated with a higher completion rate. All complications were associated with endoscopic treatment and the rate of bleeding needing hemostasis and perforation needing surgical repair were both 0.16% after colonoscopy with polypectomy. The adenoma detection rate at the first colonoscopy was 25%. Although there was no difference in the completion and complication rates based on differences in the colonoscopy experience of the endoscopist, the detection rate of adenomas and intramucosal cancers was significantly higher with more experienced endoscopists. The proportion of patients developing cancer was significantly higher with a >24 months than a ≤24 months interval.

**Conclusion:**

High‐volume experienced endoscopists and appropriate surveillance intervals may minimize the risk of developing colorectal cancers in patients with Lynch syndrome.

## INTRODUCTION

Lynch syndrome is a hereditary autosomal dominant disease that is caused by pathogenic germline variants in one of the DNA mismatch repair genes or deletions in the 3′ ends of the *EpCAM*, leading to epigenetic silencing of the *MSH2*. Affected family members have an increased risk of the development of malignancies including colorectal cancer (CRC) and endometrial cancer.^1^ The risk of development of metachronous CRC after segmental colectomy for primary CRC has been reported to be approximately 28% with a median follow‐up of 93 months.^2^

Colorectal adenomas are recognized as precursors to CRC in patients with Lynch syndrome. Regular colonoscopy surveillance provides the opportunity to prevent the development of CRC by detection and endoscopic removal of adenomas. Current guidelines recommend that patients with Lynch syndrome should have colonoscopy surveillance every 1–2 years starting at age 20–25 years.^3^, ^4^, ^5^, ^6^ A study of interval cancers in patients with Lynch syndrome showed that factors associated with the development of interval cancers included incomplete colonoscopy image, insufficient bowel preparation, and incomplete removal of adenomas during the previous examination.^7^

In recent years, several quality parameters, such as colonoscopy completion rate, adenoma detection rate (ADR), and colonoscopy‐related complication rate, have been proposed to optimize colonoscopy examination.^8^, ^9^ Evaluations of the quality of colonoscopy are routinely published and are directed toward the complete examination and removal of all polyps in patients with Lynch syndrome. In the present study, we evaluated the quality and safety of colonoscopy surveillance using data from a nationwide Japanese multicenter study.

## METHODS

Patients with a confirmed genetic diagnosis of Lynch syndrome were retrospectively registered in a nationwide Japanese multicenter study conducted by the Committee of Hereditary Colorectal Cancer of the Japanese Society for Cancer of the Colon and Rectum. Patients who underwent at least one colonoscopy were included in this study. Colonoscopy completion rate, colonoscopy‐related complication rate, proportion having an adequate colonoscopy interval, and ADR were examined. The results for these patients were compared based on the experience of the endoscopists. In addition, the frequency of developing adenomas and CRC during follow‐up was compared based on the colonoscopy interval in the 276 patients who had at least two colonoscopies. Colonoscopy was performed using white‐light endoscopy with or without magnification or dye‐based chromoendoscopy. Colonoscopy was considered complete with visualization of the ileocecal valve or appendicular orifice, intubation of the terminal ileum, or visualization of the anastomosis. Colonoscopy interval was divided into <12 months, 12–24 months, and >24 months groups. The experience of each endoscopist was categorized into two levels (> 5000 and < 5000) according to their cumulative number of colonoscopies performed. The cecum and ascending and transverse colon were considered proximal colon, whereas the descending, sigmoid colon, and rectum were considered distal colon.

All polyps were characterized according to the Paris classification.^10^ Polyps detected during the colonoscopy were removed and endoscopic treatment was performed at the discretion of the endoscopist. Histological diagnoses were made according to the classification of the Japanese Society for Cancer of the Colon and Rectum.^11^ Serrated lesions included hyperplastic polyps, sessile serrated adenomas/polyps, and traditional serrated adenomas classified according to the World Health Organization histological criteria.^12^ ADR was defined as the number of colonoscopies at which one or more adenomas were found divided by the total number of colonoscopies performed. Intramucosal cancer classified as stage 0 in the Japanese classification of colorectal carcinoma was categorized as a ‘malignancy’ and was not considered for determination of the ADR.

This study was approved by the Institutional Review Board of each participating institution and the Japanese Society for Cancer of Colon and Rectum and was performed in accordance with the provisions of the Declaration of Helsinki.

## STATISTICAL ANALYSIS

The association between two categorical variables was tested using the Chi‐squared test or Student's t‐test. Variables with a *p*‐value < 0.05 in the univariate analysis were further evaluated in multivariate analysis using a logistic regression model. SPSS version 27 (SPSS Statistics for Windows; IBM, Armonk, NY) was used for analysis. *p*‐values < 0.05 were considered significant.

## RESULTS

### Patient demographic data and details of colonoscopy

Patients with Lynch syndrome (*n* = 316) with confirmed pathogenic germline variants in mismatch repair genes from 13 institutions who underwent colonoscopy were enrolled in this study. Seven patients were excluded due to a lack of information regarding colonoscopy. The remaining patients (*n* = 309) who underwent 1749 colonoscopies were enrolled in the final analysis. Patient demographics and details of colonoscopy per patient are summarized in Table 1. The median age at first colonoscopy was 50 years (range 14–86). The following pathogenic germline variants were identified: *MLH1* (*n* = 119, 38%), *MSH2* (*n* = 138, 44%), *MSH6* (*n* = 36, 12%), *PMS2* (*n* = 11, 4%), and *EPCAM* (*n* = 5, 2%). The median follow‐up interval between the first and last colonoscopies was 7 years (range 0–25). There were 236 patients who underwent CRC surgery. Of these, 133 (43%) patients had a previous history of CRC surgery prior to the first colonoscopy, 95 (31%) patients were diagnosed with CRC at the first colonoscopy and underwent CRC surgery, and 25 patients underwent CRC surgery during follow‐up. The median number of colonoscopies was five (range 1–18). The percentage of patients with all colonoscopies complete was 94% (291/308). The percentage of patients who underwent colonoscopy with all intervals within 2 years was 76% (211/276), and 71% (196/276) of patients had all colonoscopies complete with all intervals within 2 years. Endoscopic treatment was performed in 219 (71%) patients. The median number of endoscopic treatments per patient was two (range 1–43). The ADR at the first colonoscopy was 25% (78/309) and in patients without previous CRC surgery was 22% (39/176). The adenoma and intramucosal cancer detection rate at the first colonoscopy was 34% (106/309). The number of patients who had at least one or more adenoma, intramucosal cancer, or serrated polyps during the follow‐up interval was 175 (57%), 95 (31%), and 86 (28%), respectively. There were four patients with complications (perforation = 1, bleeding = 2, and abdominal discomfort = 1) in the present study. The patient with abdominal discomfort during endoscopic mucosal resection needed discontinuation of treatment. One of two patients with bleeding needed endoscopic hemostasis. The last patient with perforation needed emergency surgical intervention. All were associated with endoscopic treatment.

**TABLE 1:** Patient demographic data and details of colonoscopy

**Patients with Lynch syndrome**	**309**
Median age at first colonoscopy (range)	50 (14–86)
Sex—Male:Female	144:165
Presence of causative genes of Lynch syndrome	
*MLH1*	119 (38%)
*MSH2*	138 (44%)
*MSH6*	36 (12%)
*PMS2*	11 (4%)
*EPCAM*	5 (2%)
Median follow‐up between first and last colonoscopy, years (range)	7 (0–25)
Patients undergoing surgery for CRC	236
Patients with previous CRC surgery prior to the first colonoscopy	133 (43%)
Patients without previous CRC surgery during follow‐up	116
Patients diagnosed with CRC with surgery during follow‐up	17
Patients without previous CRC surgery prior to the first colonoscopy	103 (33%)
Patients diagnosed with CRC at the first colonoscopy who underwent surgery	95
Patients with CRC surgery during follow‐up	8
Colonoscopy	
Median number of colonoscopies (range)	5 (1–18)
Percentage of patients with all colonoscopies complete	94% (291/308^†^)
Percentage of patients with ≤ 24 months interval between colonoscopies	76% (211/276^‡^)
Percentage of patients without incomplete colonoscopy and >24 months interval between colonoscopies	71% (196/276^‡^)
Endoscopic treatment	
Underwent endoscopic treatment	219 (71%)
Median number of endoscopic treatments/patient (range)	2 (1–43)
Adenoma detection rate at first colonoscopy	25% (78/309)
Adenoma and intramucosal cancer detection rate at first colonoscopy	34% (106/309)
Adenoma detection rate at first colonoscopy in patients without previous CRC surgery	22% (39/176)
Patients with one or more polyps resected by endoscopic treatment during follow‐up	
With adenoma	175 (57%)
With intramucosal cancer	95 (31%)
With serrated polyps	86 (28%)
Complications related to endoscopic treatment	
Any complication	4 (1.2%)
Bleeding necessitating repeat colonoscopy	2 (0.6%)
Bleeding necessitating endoscopic hemostasis	1 (0.3%)
Perforation needing emergency surgical intervention	1 (0.3%)

^†^ Excluding one patient who underwent sigmoid colonoscopy without preparation.

^‡^ Excluding patients who had only one colonoscopy.

Abbreviation: CRC, colorectal cancer.

### Clinical characteristics for each colonoscopy

The clinical characteristics of colonoscopies are shown in Table 2. The colonoscopy completion rate was 98.8% (1630/1649). The completion rate was significantly lower in colonoscopies of patients who had not undergone CRC surgery (97.1% [501/516]) compared with those who had undergone surgery (99.6% [1129/1133]; *p* < 0.001). Colonoscopy intervals of more than 24 months occurred in 6% (78/1397). Endoscopic treatment was performed during 634 (36%) colonoscopies and 1184 polyps were resected. The mean number of resected polyps per colonoscopy was 0.68 ± 1.2. The following pathologies were identified: adenoma (*n* = 807), intramucosal cancer (*n* = 191), and serrated polyp (*n* = 157). The ADR and the adenoma and intramucosal cancer detection rates in all examinations were 26% (462/1749) and 32% (558/1749), respectively. The occurrence of complications related to endoscopic treatment was four. The rate of needing both endoscopic hemostasis and emergency surgery was 0.06% (1/1652).

**TABLE 2:** Clinical characteristics of each colonoscopy

**Total number of colonoscopies**	**1749**
Colonoscopy completion rate	98.8% (1630/1649^†^)
Completion rate in patients without previous CRC surgery	97.1% (501/516)
Completion rate in patients with previous CRC surgery	99.6% (1129/1133)
Colonoscopy interval^‡^	
<12 months	383
12–24 months	936
>24 months	78
Endoscopic treatment	
Colonoscopy with endoscopic treatment (%)	634 (36%)
Resected polyps	1184
Median number of resected polyps/colonoscopy (range)	0 (1‐10)
Mean number of resected polyps/colonoscopy	0.68 ± 1.2
Pathology of polyps resected endoscopically	
Adenoma	807
Intramucosal cancer	191
Serrated lesions	157
Others	33
Adenoma detection rate among all colonoscopies	26% (462/1749)
Adenoma and intramucosal cancer detection rate among all colonoscopies	32% (558/1749)
Complications related to endoscopic treatment	
Colonoscopies with any complication related to endoscopic treatment	0.2% (4/1652^§^)
Colonoscopies with bleeding needing repeat colonoscopy	0.1% (2/1652^§^)
Colonoscopies with bleeding needing endoscopic hemostasis	0.06% (1/1652^§^)
Colonoscopies with perforation needing emergency surgical intervention	0.06% (1/1652^§^)

^†^ excluding colonoscopy for purpose of endoscopic treatment, preoperative marking and examination of anastomosis, colonoscopy without preparation, colonoscopy for patients with stenosis due to cancer, colonoscopy for patients who underwent total colectomy, and sigmoidoscopy.

^‡^ intervals were examined among patients who underwent colonoscopy more than two times.

^§^ colonoscopies with information of complication.

Abbreviation: CRC, colorectal cancer.

### Complications related to endoscopic treatment

The rate of bleeding requiring endoscopic hemostasis was 0.16% (1/607) per colonoscopy with endoscopic treatment (Table 3). The rate of perforation needing surgical repair was 0.16% (1/607) for colonoscopies performed with endoscopic treatment. Perforation occurred during endoscopic mucosal resection of a 25‐mm 0‐IIa intramucosal carcinoma in the transverse colon after right hemicolectomy. Bleeding occurred after endoscopic snare polypectomy of a 6‐mm 0‐IIa intramucosal carcinoma in the transverse colon after partial resection of the sigmoid colon. The frequencies of bleeding and perforation were 0.66% (1/152) and 0.66% (1/152) for colonoscopies performed with endoscopic treatment for intramucosal cancer, respectively.

**TABLE 3:** Complications related to endoscopic treatment

**Complications**	**Frequencies**
Any complication related to endoscopic treatment	0.2% (4/1652^†^)
Bleeding after endoscopic treatment needs endoscopic hemostasis	
Bleeding among all colonoscopy procedures	0.06% (1/1652)
Bleeding among all colonoscopies with endoscopic treatment	0.16% (1/607)
Bleeding among all colonoscopies with endoscopic treatment for intramucosal cancer	0.66% (1/152)
Perforation after endoscopic treatment needing emergency surgery	
Perforation among all colonoscopy procedures	0.06% (1/1652)
Perforation among all colonoscopies with endoscopic treatment	0.16% (1/607)
Perforation among all colonoscopies with endoscopic treatment for intramucosal cancer	0.66% (1/152)

^†^ colonoscopies with information of complication.

### Colon polyp detection and colonoscopy intervals

The relationship between the development of polyps and the colonoscopy interval (12–24 months: 936 colonoscopies and >24 months: 78 colonoscopies) is shown in Table 4. There was no difference in the rate of development of adenomas between the two groups. The frequencies of developing intramucosal cancer and invasive cancer were significantly higher in the group with an interval >24 months (*p* = 0.01, 0.01, respectively).

**TABLE 4:** Colon polyps detected and colonoscopy intervals

	**Interval 12–24 months (936 colonoscopies)**	**Interval more than 24 months (78 colonoscopies)**	***p*‐values**
Number of polyps detected			
Adenomas	232 (25%)	16 (21%)	NS
Intramucosal cancers	42 (4.5%)	10 (13%)	0.01
Invasive cancers	9 (0.96%)	5 (6%)	0.01

Abbreviation: NS, not significant.

### Colonoscopy parameters and endoscopist's experience

Data were compared between two levels of experience of the endoscopist (Table 5). There was no difference in the completion rate or complication rate between the two groups. However, the mean number of detected adenomas and intramucosal cancers was significantly higher in colonoscopies performed by more experienced endoscopists (*p* = 0.03, *p* = 0.01, respectively).

**TABLE 5:** Colonoscopy parameters and the endoscopist experience

	**5000 or more**	**<5000**	***p*‐values**
Completion rate	98.4% (718/730)	99.2% (900/907)	NS
Colonoscopy‐related complication rate	0.28% (2/720^†^)	0.24% (2/823^†^)	NS
Mean number of detected adenomas	0.54	0.40	0.03
Mean number of detected intramucosal cancers	0.15	0.08	0.01
Mean number of detected serrated polyps	0.09	0.09	NS

^†^ colonoscopies with information of complication.

Abbreviation: NS, not significant.

### Multivariate analysis for the detection of intramucosal cancer

Interval time and the endoscopist's skill were significantly associated with the detection of intramucosal cancer in each analysis (Tables 4 and 5). They were further evaluated by multivariate analysis using a logistic regression model. Both of these independent factors were significantly associated with the detection of intramucosal cancer (Table 6).

**TABLE 6:** Multivariate analysis for detection of intramucosal cancer

	**Detection of intramucosal cancer**		
	***p*‐values**	**Odds ratio**	**95% CI**
Colonoscopy intervals			
(12–24 months vs. more than 24 months)	0.01	3.40	1.62–7.14
Endoscopist's experience			
(< 5000 vs. 5000 or more)	0.02	1.98	1.12–3.52

Abbreviation: 95% CI, 95% confidence interval.

## DISCUSSION

This study assessed the quality and safety of colonoscopy surveillance for patients with Lynch syndrome registered in a nationwide Japanese multicenter study. The colonoscopy completion rate was 98.8% per colonoscopy and history of CRC surgery was significantly associated with a higher completion rate. Seventy‐one (196/276) percent of patients underwent complete colonoscopies with adequate intervals for all colonoscopies. All complications were associated with endoscopic treatment, and the rates of bleeding necessitating hemostasis and perforation needing surgical intervention were both 0.16% for colonoscopies performed with endoscopic treatment. The ADR and adenoma and intramucosal cancer detection rates at the first colonoscopy were 25% (78/309) and 34% (106/309) among all patients, respectively. Although there was no difference in the completion and complication rates according to the experience level of the endoscopist, the detection rate of adenoma and intramucosal cancer was significantly higher among more experienced endoscopists. The proportion of intramucosal cancer or invasive CRCs detected was significantly higher for patients with an interval of more than 24 months compared to less than 24 months. This was the first study to assess the current quality and safety of colonoscopy surveillance for patients with Lynch syndrome in a nationwide survey in Japan.

Colonoscopy has variable quality depending on the endoscopist's skill and the patient's background. In the management of Lynch syndrome, patients are younger and repeated colonoscopies at short intervals are needed for lifelong surveillance. Moreover, colonoscopy in patients with Lynch syndrome should be precisely performed with special attention to the right colon. Thus, the colonoscopy completion rate is an important quality parameter in such patients. In the present study, the completion rate was 98.8% per colonoscopy, similar to that reported (92%–99%) in a previous study.^13^, ^14^, ^15^ The completion rate was significantly lower in patients without a history of prior surgery (97.1%) compared with those with a history of surgery (99.6%) (*p* < 0.001). Although adhesions associated with previous abdominal surgery decrease the completion rate,^16^ the reduction of the length of the colon due to a past history of CRC surgery may be associated with an increased completion rate in patients with Lynch syndrome.

The rates of bleeding and perforation during colonoscopy performed with and without endoscopic treatment are considered to be quality and safety indicators in some guidelines.^17^ ^.^ ^18^ The rate of post‐polypectomy bleeding and perforation is recommended to be ≤1/100 and ≤1/500 colonoscopies.^17^, ^18^, ^19^ In the present study, the rates of bleeding and perforation for colonoscopies performed with endoscopic treatment were both 0.16% (1/607), which is within the acceptable range. Both of the polyps associated with complications were flat‐type intramucosal cancers in the transverse colon. Complications related to endoscopic treatment tended to occur with large, flat polyps.^20^ Clipping has been used recently to prevent or treat bleeding or perforation. The rates of bleeding and perforation in the present study were consistent with the proposed standard. Thus, colonoscopy surveillance with endoscopic treatment was performed safely for patients with Lynch syndrome.

Colorectal adenomas are recognized as the main precursor to CRC in patients with Lynch syndrome. The efficacy of colonoscopy screening is based on the concept of a “clean” colon by removing all of the adenomas identified. The ADR is a widely accepted benchmark for the quality of screening colonoscopy.^21^ ADRs in colonoscopy surveillance using standard white light have been reported to be 25%–35% for the general population^22^, ^23^, ^24^, ^25^ and 20%–31% for patients with Lynch syndrome.^14^, ^15^, ^26^, ^27^ ADRs for patients with Lynch syndrome are not so high compared to those of the general population. In the present study, the ADR and adenoma, and intramucosal cancer detection rates at the first colonoscopy were 25% (78/309) and 35% respectively, which are comparable with results in previous reports. The ADR in the present study was based mainly on white‐light endoscopy. Recent advanced imaging techniques, such as virtual chromoendoscopy, improve the ADR.^26^ There is evidence that with each 1.0% increase in ADR, there is an associated 3.0% decrease in the risk of developing an interval CRC.^28^, ^29^ The European Society of Gastrointestinal Endoscopy guideline recommends at least the use of a high‐definition endoscope in patients with Lynch syndrome.^30^ The quality of colonoscopy in the present study was acceptable but using more advanced colonoscopy techniques may improve ADR in patients with Lynch syndrome.

In the present study, although there was no difference in completion and complication rates according to the level of experience of the endoscopist, the number of detected adenomas and intramucosal cancers was significantly higher in colonoscopies performed by more experienced endoscopists. A population‐based study from Canada found that the risk of complications, such as perforation and bleeding, was increased threefold in procedures performed by endoscopists who had performed fewer than the threshold of 300 colonoscopies per year.^31^ In addition, the ADR was positively associated with the level of experience of the endoscopist.^32^ A minimum lifetime experience of 1000 examinations and a minimum annual number of 150 screening colonoscopies is recommended by the English NHS Bowel Cancer Screening Program.^33^ High‐volume experienced endoscopists may have an important role in minimizing the number of missed polyps.

Several factors may predict failure to prevent the development of cancer during follow‐up in patients with Lynch syndrome. The adenoma‐carcinoma sequence appears to be accelerated in patients with Lynch syndrome, with the polyp to cancer dwell time estimated at 35 months compared with 10–15 years for sporadic cancers.^34^ The post‐colonoscopy risk for developing CRC was significantly lower in carriers receiving high‐quality surveillance.^15^ Strict annual or biennial surveillance may minimize the risk of the development of CRC. In the present study, 24% (65/276) of patients underwent at least one colonoscopy with an inadequate interval of >24 months. The proportion of patients developing intramucosal and invasive CRC was significantly higher in patients with an inadequate interval compared to those with an adequate interval. In the general population, a 10‐year colonoscopy interval is considered sufficient to successfully lower the incidence of CRC.^35^ However, in patients with Lynch syndrome, the progression to CRC has been suggested to be accelerated compared to the general population.^36^ Appropriate surveillance intervals for complete colonoscopy may minimize the risk of developing CRC.

There were several limitations to the present study. First, this was a retrospective study; however, a strength of this study was that the data were derived from a Japanese nationwide multicenter study of the largest Japanese cohort of patients with Lynch syndrome. Second, information regarding bowel preparation and withdrawal time as indicators of quality was lacking. These factors affected completion and ADR. Third, the study lacked sufficient data for the use of high‐definition colonoscopy and dye‐based chromoendoscopy. These advanced colonoscopy techniques may improve ADR. Finally, the study lacked detailed information about the kind of endoscopic treatment employed such as cold polypectomy, hot polypectomy, endoscopic mucosal resection, or endoscopic submucosal dissection. This information helps to delineate the precise quality and safety of endoscopic treatment for colorectal polyps in patients with Lynch syndrome.

In conclusion, the present study reports the current status of quality and safety of colonoscopy surveillance for patients with Lynch syndrome in a nationwide survey in Japan. These findings suggest that high‐volume experienced endoscopists and appropriate surveillance intervals may minimize the risk of developing CRC in patients with Lynch syndrome.

## CONFLICT OF INTEREST

The authors declare that they have no conflict of interest.

## FUNDING INFORMATION

None.
